# Identification of Novel Growth Factor Conjugated Nanofibers for Stimulation of Neuronal Growth

**DOI:** 10.1002/mabi.202500585

**Published:** 2026-01-23

**Authors:** Yu‐Liang Tsai, Karla K. Rivera, Nayeong Jeon, Bernd Knöll, Christopher V. Synatschke

**Affiliations:** ^1^ Max Planck Institute For Polymer Research Mainz Germany; ^2^ Institute of Neurobiochemistry Ulm University Ulm Germany

**Keywords:** FGF, growth factor, IGF, nanofiber, neurite, neuron, regeneration, self assembling peptide

## Abstract

Growth factors (GF) fulfil essential functions during organ development and regeneration. In tissue regeneration, evidence suggests that the combined application of several GFs is more efficient compared to their individual application. Single or multiple GFs are often applied to animal models of organ regeneration through release by hydrogels. Such hydrogels are often formed by self‐assembling peptides (SAPs) spontaneously polymerizing into peptide nanofiber (PNF) networks. In this study, we established PNFs by conjugating an SAP backbone (KIKIQIN) with bioactive peptide sequences derived from two GFs, FGF2 and IGF1. This resulted in the GF‐mimicking fusion peptides FGF2‐SAP and IGF1‐SAP, respectively. In these PNFs, both GFs were stably incorporated rather than released as in the case of PNF‐derived hydrogels. When individually added to culture medium, FGF1‐SAP and IGF1‐SAP stimulated the growth of mouse primary hippocampal neurons. Notably, their growth‐stimulating potential exceeded neuronal growth achieved with the SAP backbone or the GF peptides alone. Finally, combinations of FGF2‐SAP, IGF1‐SAP, and the SAP backbone were tested, which formed FGF2 and IGF1 presenting PNFs. Indeed, specific GF‐SAP combinations resulted in elevated numbers of surviving neurons compared to individual application. In summary, in this study, we identified novel GF‐SAP hybrid nanofibers capable of stimulating cellular growth. Such nanofibers, enabling stable and simultaneous presentation of multiple GFs, might be well suited for tissue regeneration in vivo.

## Graphical abstract

In this study, novel hybrid nanofibers composed of self‐assembling peptides (SAPs) conjugated with peptides derived from two growth factors (GFs), FGF2 and IGF1, were produced (FGF2‐SAP and IGF1‐SAP). Those hybrid nanofibers stimulated cellular growth of primary mouse neurons. In addition, nanofibers simultaneously presenting both GFs also enhanced neuronal growth. Such nanofibers might be well suited for tissue regeneration in vivo.

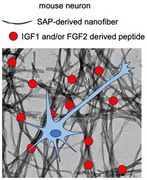

## 1 Introduction

Growth factors (GFs) regulate diverse cellular functions during development and in the maintenance of physiological organ homeostasis, including proliferation, growth, and differentiation. In the context of tissue repair in injuries such as spinal cord injury or traumatic brain injury, single or combined GF administration is studied in animal models; however, these approaches have not yet been established as therapeutic options for patients [1, 2]. GFs are secreted by cells; however, they are frequently sequestered in the extracellular matrix (ECM), resulting in GF gradients and high local GF concentrations [3]. In biomaterials research, GFs are commonly incorporated into hydrogels and transplanted to the injury site [4], which results in GF delivery over a defined period. However, due to short GF half‐lives, the biological activity of single GFs or GF cocktails remains limited. One way to circumvent this problem is by using short GF peptides derived from full‐length GFs, which have a similar potential to activate GF receptors and downstream signaling. Such GF mimetic peptides bear many of the activities of the full‐length growth factors, but they do not have an affinity as a binding protein to the GF itself. Recently, such GF‐mimetic peptides have been identified for several GFs, including FGF2 [5] and IGF1 [6]. For instance, a 15 amino acid sequence (YRSRKYSSWYVALKR) derived from full‐length FGF2 binds heparin, interacts with FGFR1, and promotes cell adhesion [7]. Recently, this FGF‐mimetic peptide was incorporated in a self‐assembling peptide (SAP) amphiphile, forming peptide nanofibers (PNFs). The resulting bioactive scaffold promoted migration and proliferation of endothelial cells [8] and stimulated recovery from spinal cord injury in a rodent model [9].

Similarly, a 12‐amino acid‐derived IGF1 stretch (GYGSSSRRAPQT) is capable of activating the IGF1 receptor and mimicking full‐length IGF1 activity, particularly when fused to an SAP sequence [6, 10]. In vivo, hydrogels of this IGF1‐SAP mimetic peptide improved ischemia and spinal cord injury recovery in animal models [6, 10]

In many types of organ regeneration, application of GF mixtures has been shown to be superior to individual application [11], as demonstrated in spinal cord injury repair [12]. Thus, in this study, we aimed to establish the synthesis of SAP‐derived PNFs presenting the two GF‐derived mimetics of FGF2 and IGF1 (labelled FGF2‐SAP6 and IGF1‐SAP6, respectively). Previously, hydrogels encapsulating multiple bioactive molecules, including GFs and ECM‐derived epitopes, enhanced tissue regeneration in models of peripheral nerve or skin injury [13, 14, 15]. However, since hydrogel swelling, bioactivity, biocompatibility, and degradability [16] often affect their function in the injury site and undergo rapid degradation, we sought to establish an approach where multiple GFs are covalently conjugated to SAPs, forming PNFs capable of providing more sustained GF activity. For this, primary mouse neurons labelled with βIII tubulin as an in vitro model of neuronal growth have been employed. Full‐length FGF2 and IGF1 are well‐established stimulators of neuronal growth [17, 18]. Here, we show that the two GF mimetics, FGF2 or IGF1, fused to an SAP sequence (KIKIQIN), formed PNFs and elevated neuronal growth compared to the GF derived peptides and the SAP sequence alone. Furthermore, we prepared PNFs simultaneously presenting FGF2 and IGF1, which improved the number of surviving neurons compared to FGF2‐SAP or IGF1‐SAP alone.

Thus, in this study, we present a nanofiber‐based approach allowing for the presentation of desired GF combinations, offering the potential for designing tissue‐specific regeneration responses.

## 2 Materials and Methods

### 2.1 Materials

Fluorenylmethyloxycarbonyl (Fmoc) protected amino acids and ethyl cyano(hydroxyimino) acetate (Oxyma Pure) were purchased from Novabiochem. Fmoc‐Asn (Trt) Wang Resin was acquired from Merck KGaA. Congo Red, α‐cyano‐4‐hydroxycinnamic acid (CHCA), N,N'‐diisopropylcarbodiimide (DIC), N,N‐diisopropylethylamine (DIPEA), dimethyl sulfoxide (DMSO), triisopropylsilane (TIPS), were bought from Sigma–Aldrich. N,N‐dimethylformamide (DMF, peptide grade), dichloromethane (DCM), fluorescamine, and 4% uranyl acetate solution were purchased from Thermo Fisher Scientific. Piperidine (peptide grade) and trifluoroacetic acid (TFA) were obtained from Carl Roth GmbH. Diethyl ether and acetonitrile (ACN, HPLC grade) were acquired from Honeywell. Peptides (purity > 95%) for initial screening (SAP1 to SAP9 listed in Table 1) were purchased (Zhengzhou Peptides Pharmaceutical Technology Co., Ltd). All chemicals were used upon arrival without further purification. Ultrapure water was purified through a Millipore purification system (Milli‐Q).

**TABLE 1:** Amino acid sequences of peptides used.

Abbreviation	Amino acid sequence
SAP1	RGDKIKIQIC
SAP2	RGDKIKIQINM
SAP3	KIKIQINM
SAP4	KIKIQINMWWWQ
SAP5	KFKFQFNMWQ
SAP6	KIKIQIN
SAP7	KIKIKIQINMWQ
SAP8	HIHIQINM
SAP9	CKFKFQF
FGF2	YRSRKYSSWYVALKR
FGF2‐SAP6	YRSRKYSSWYVALKR‐G‐SAP6
IGF1	GYGSSSRRAPQT
IGF1‐SAP6	GYGSSSRRAPQT‐G‐SAP6

### 2.2 Solid Phase Peptide Synthesis (SPPS)

All peptides were synthesized in an automated microwave peptide synthesizer (Liberty BlueTM, CEM Corporation) from C‐ to N‐terminus by Fmoc‐SPPS method using Fmoc‐Asn (Trt), Fmoc‐Arg (Pbf)‐Wang, and Fmoc‐Thr (tBu) Wang Resins with a size of 100–200 mesh. DMF was used as the main washing solvent, and the deprotection solvent consisted of 20% piperidine in DMF. The activator and the activator base were 0.5 m DIC and 1.0 m Oxyma, respectively, in DMF. The amount of materials and reagents was prepared according to the Liberty BlueTM software calculation. In brief, the resins were swollen with DMF for 30 min prior to synthesis. The procedure began with Fmoc removal, and the resins were immersed in the deprotection solvent and heated to 75°C (155 W) for 15 s and 90°C (30 W) for 50 s, followed by washing with DMF twice. Subsequently, amino acids were coupled from 0.2 m solutions of the respective amino acid in DMF, facilitated by an activator and an activator base. The reaction was heated to 75°C (170 W) for 15 s and 90°C (30 W) for 110 s, followed by flushing with DMF. The deprotection solvent was added to remove the Fmoc motif after the final coupling. Afterward, the resin beads were first flushed with DCM and immersed in a cleavage cocktail (95% TFA, 2.5% MilliQ water, 2.5% TIPS) for 2 h. Next, the beads were precipitated in cold diethyl ether and centrifuged to obtain crude peptide precipitate. The amino acids of the peptide sequences are listed in Table 1.

### 2.3 Purification and Characterization of Peptides

The peptide precipitate was solubilized in a mixture of Milli‐Q water and ACN and subsequently purified using preparative reversed‐phase high‐performance liquid chromatography (RP‐HPLC) on a Shimadzu system equipped with a Phenomenex Gemini C18 column (5 µm particle size, 110 Å, 150 × 30 mm dimensions) at a flow rate of 25 mL/min. The purification employed a solvent gradient composed of ACN and Milli‐Q water containing 0.1% TFA. Sample fractions were collected based on retention times, monitored via UV absorption at 214 nm. The purified peptides were lyophilized and stored at −20°C until further use.

Samples were analyzed using matrix‐assisted laser desorption/ionization time‐of‐flight mass spectrometry (MALDI‐ToF MS) employing the dried droplet method. Each sample was combined with a saturated solution of CHCA matrix, prepared in a 1:1 (v/v) mixture of Milli‐Q water and ACN. Mass spectra were acquired using a rapifleX MALDI‐ToF/ToF mass spectrometer (Bruker).

The purity of the samples was assessed using analytical RP‐HPLC (Shimadzu), equipped with a ZORRBAX Eclipse XDB‐C18 column (5 µm, 70 Å, 9.4 × 250 mm) at a flow rate of 4 mL/min. A gradient of solvent mixture of ACN/MilliQ with 0.1% TFA began from 5% to 80% ACN. The samples were monitored at 214 nm UV absorption.

### 2.4 Preparation and Characterization of SAPs

#### 2.4.1 Self‐Assembly

Each peptide powder was dissolved in DMSO (10 mm stock solution) and further diluted into PBS (phosphate‐buffered saline without calcium chloride and magnesium chloride, Thermo Fisher Scientific) to obtain the incubation concentration of 500 µm at room temperature for 24 h, unless otherwise stated.

#### 2.4.2 Fluorescence Based Conversion Rate (CR) Assay

The amount of peptide monomers assembled into nanostructures was assessed by a CR assay based on a previous report [19]. In short, 200 µL of 1 mm peptide solution was incubated for 24 h, and thereafter 100 µL of peptide solution was centrifuged in a Vivaspin 500 centrifugal concentrator (3 kDa) to separate fibers and peptide monomers at a rate of 12 000 × g for 60 min at 4°C. Both filtrated and non‐filtrated solutions were lyophilized and dissolved in 30 µL of DMSO. Subsequently, 9 µL of the amine‐reactive dye fluorescamine (10 mg/mL in DMSO) was added to the filtrated and non‐filtrated ones, followed by incubation in a 384‐well plate (black UV Star, GreinerBio‐One) at room temperature for 20 min. Fluorescence was recorded on a microplate reader (Spark, Tecan) with excitation at λ = 365 nm and emission at λ = 470 nm (bandwidth = 10 nm and multiple readings per well, 3 × 3). The conversion rate is calculated using the following equation. Each measurement was performed in triplicate and plotted with Prism Software.

$$\begin{array}{ccc} \mathrm{Cr} & = & [1-((\mathrm{filtrated}\;\mathrm{fluorescence}\;\mathrm{intensity})/\\ & & (\mathrm{nonfiltrated}\;\mathrm{fluorescence}\;\mathrm{intensity}))]\% \end{array}$$

### 2.5 Zeta Potential (ζ)

Zeta‐potential measurements were conducted using a Zetasizer Nano ZS (Malvern Instruments) to determine the surface charge of aggregated peptides following a previous report [19]. Peptide solutions (60 µL) were diluted in 600 µL of 1 mm KCl solution in 1 mL disposable capillary cells (DTS1060, Zetasizer Nano series, Malvern Instruments). Each measurement was performed in triplicate and plotted with Prism Software.

### 2.6 Transmission Electron Microscopy (TEM)

TEM samples were prepared by depositing 5 µL of peptide solution on copper grids coated with carbon and formvar layer (300 mesh, Plano GmbH) for 10 min. The grids were stained with 4% uranyl acetate solution for 3 min, washed three times, and dried with filter paper. Measurements were performed on a Jeol 1400 electron microscope with 120 kV acceleration voltage to observe morphologies. Images were processed with ImageJ [20].

### 2.7 Attenuated Total Reflectance FT–IR (ATR‐FTIR) Spectroscopy

Each peptide solution (200 µL) was lyophilized into powder before measurement. All spectra were acquired with a resolution of 4 cm^−1^ and 64 scans on a Bruker Tensor27 spectrometer (Bruker) with a diamond crystal as ATR element (PIKE MiracleTM FTIR). The data were plotted with Origin Software.

### 2.8 Molecular Binding Assay

Proteostat Assay was prepared according to the manufacturer's instructions (ProteoStat Aggresome Detection Kit, Enzo Life Sciences). In brief, 1 µL of diluted Proteostat solution (100‐fold from Protestat solution stock in DPBS) was added to 9 µL of peptide solution and a blank solution (9 µL of DPBS with 10%v/v DMSO). Subsequently, the solution mix was deposited in a black 384‐microliter well plate (Greiner Bio‐One) for 10 min of incubation. The fluorescence emission was recorded at λem = 600 nm upon excitation at λex = 500 nm.

The Thioflavin T (ThT) assay was performed based on a previous report [19]. 5 µL of peptide solution and a blank (DPBS with 10%v/v DMSO) were mixed with ThT solution (20 µL, 50 µm) in a black 384 microliter well plate (Greiner bio‐one) and incubated for 15 min. The fluorescence emission was recorded at λem = 488 nm upon excitation at λex = 440 nm.

Peptides were considered as Proteostat‐active and‐ThT active if the fluorescence intensity was at least twice as strong compared to the control (DPBS with 10% v/v DMSO) [15]. Fluorescence spectra were recorded on a microplate reader (Spark, Tecan) with a bandwidth = 10 nm and multiple readings per well (3 × 3). The data were plotted by Prism.

### 2.9 Primary Neuronal Cultures

C57BL/6J pups (post‐natal day 0–2) were sacrificed by decapitation directly into ice‐cold PBS. All animal experiments were approved by the local ethics committee (Regierungspräsidium Tübingen, Tübingen, Germany; number: O.181‐9).

The hippocampi were isolated in ice‐cold HBSS, then digested in 0.05% Trypsin‐EDTA for 10 min at 37°C. Following digestion, tissues were washed twice with pre‐warmed HBSS, and subsequently incubated in 1 mL DMEM supplemented with 10% fetal bovine serum (FBS) to inactivate trypsin. Tissues were gently dissociated with a full‐diameter, flame‐polished Pasteur pipette tip, followed by a half‐diameter, flame‐polished pipette tip for 1 min each. The resulting cell suspension was spun down at 600 × g for 7 min. After removing the supernatant, the cell pellet was resuspended in fresh culture media. Cells were counted with a Neubauer chamber and plated at a density of 5000 cells per well in 96‐well glass‐bottom plates (P96‐1.5H‐N, Celvis). Cultures were maintained in a humidified incubator kept at 37°C and 5% CO_2_ and stained after 24 h in culture for all experiments.

### 2.10 Peptide Preparation

SAPs underwent fiber formation in aqueous solutions. To prevent premature assembly, the synthesized SAP powders were dissolved in 99.9% dimethyl sulfoxide (DMSO) to create 10 mg/mL stock solutions, which were stored at −20°C. For fiber formation, stock solutions were diluted to 1 mg/mL in PBS and incubated at room temperature for 16 h. For coating experiments, peptide solutions were further diluted the following day with PBS to a final concentration of 50 µg/mL and used to coat 96‐well glass‐bottom plates. The solutions were left on the plates overnight at 37°C and 5% CO_2_ and washed once with ddH_2_O. Concurrently, some wells were left as pure glass (“blank”, “uncoated”) to serve as a negative control, or coated with poly‐L‐lysine (P6282, Sigma–Aldrich) and mouse laminin (L2020, Sigma–Aldrich) to simulate optimal growing conditions. For these, wells were coated with 100 µg/mL poly‐L‐lysine (PLL) for 1 h at 37°C and 5% CO_2_, before being washed 3× with ddH_2_O, followed by 20 µg/mL laminin overnight at 37°C and 5% CO_2_. The following day, the laminin was removed, and the wells were washed 3× with HBSS. Hippocampal neurons were plated into all wells at a density of 5000 cells per well directly with culture media.

Similar to the SAPs, the growth factor‐conjugated peptides were maintained as 10 mg/mL stock solutions in DMSO at −20°C. For fiber assembly, stock solutions were diluted to 1 mg/mL in double‐distilled water (ddH_2_O) and incubated overnight at room temperature. The following day, the peptides were diluted to their final working concentrations and added directly into neuronal cell media (experiments to Figures 4, 5, 6).

For co‐assembly experiments, stock solutions of FGF2‐SAP6 and IGF1‐SAP6 were combined in varying ratios totaling 50% of the final peptide content, while the corresponding SAP6 without the GF‐mimetic sequence was maintained as the other 50%. The mixtures were diluted to a concentration of 1 mg/mL in PBS and incubated overnight at room temperature for fiber formation. The following day, the peptides were diluted to their final working concentrations using neuronal cell media.

### 2.11 Immunocytochemistry

Cells were washed with ice‐cold PBS and fixed in 4% PFA + 5% glucose in PBS for 15 min at room temperature. After washing three times with PBS, cells were permeabilized with 0.1% Triton‐X‐100 in PBS for 5 min. Cells were washed two more times in PBS and allowed to block for 30 min in 2% BSA in PBS before incubating with β‐tubulin III (0.28 µg mL‐1, PRB‐435P, TUJ1; Covance) for 1 h at room temperature. Cells were washed 3 times with PBS and then incubated for 1 h in the dark at room temperature with Alexa Fluor 488 donkey anti‐rabbit (2 µg/mL, A21206, Thermo Fisher) and Phalloidin Texas Red (1U/mL, #00033, Biotium). Cells were washed twice with PBS and incubated for 5 min with DAPI (1:2000 dilution in PBS). DAPI was replaced with 1% PFA in 1.25% sucrose in PBS. Plates were sealed with parafilm and stored at 4°C.

### 2.12 Quantification and Statistics

Plates were scanned with an Olympus IX‐83 microscope using the scanR 3.1 Acquisition Software. Images were analyzed using a neural network designed and provided by Evident Scientific using the scanR 3.1 Analysis Software. For quantification of neuron numbers/area, each neuron was grouped into one of three size categories (small: 80–250 µm^2^; medium: 251–500 µm^2^; large: > 501 µm^2^). After that, the neuron numbers for each of these three categories were depicted on the graphs (e.g., Figure 3K). Neurite junctions reflect the points where two neurite branches were formed. The number of such junctions/neuron were quantified in Figure 6J.

Statistical analysis was conducted using the Kruskal–Wallis test with Dunn's multiple comparisons, or one‐way ANOVA with Tukey's or Holm‐Šídák's multiple comparisons tests, depending on normality and homogeneity of variance. Significance is denoted as ^*^ *P*≤0.05, ^**^ *P*≤0.01, ^***^ *P*≤0.001.

## 3 Results

### 3.1 Physical and Biological Characterization of SAP Backbones

**FIGURE 1:**
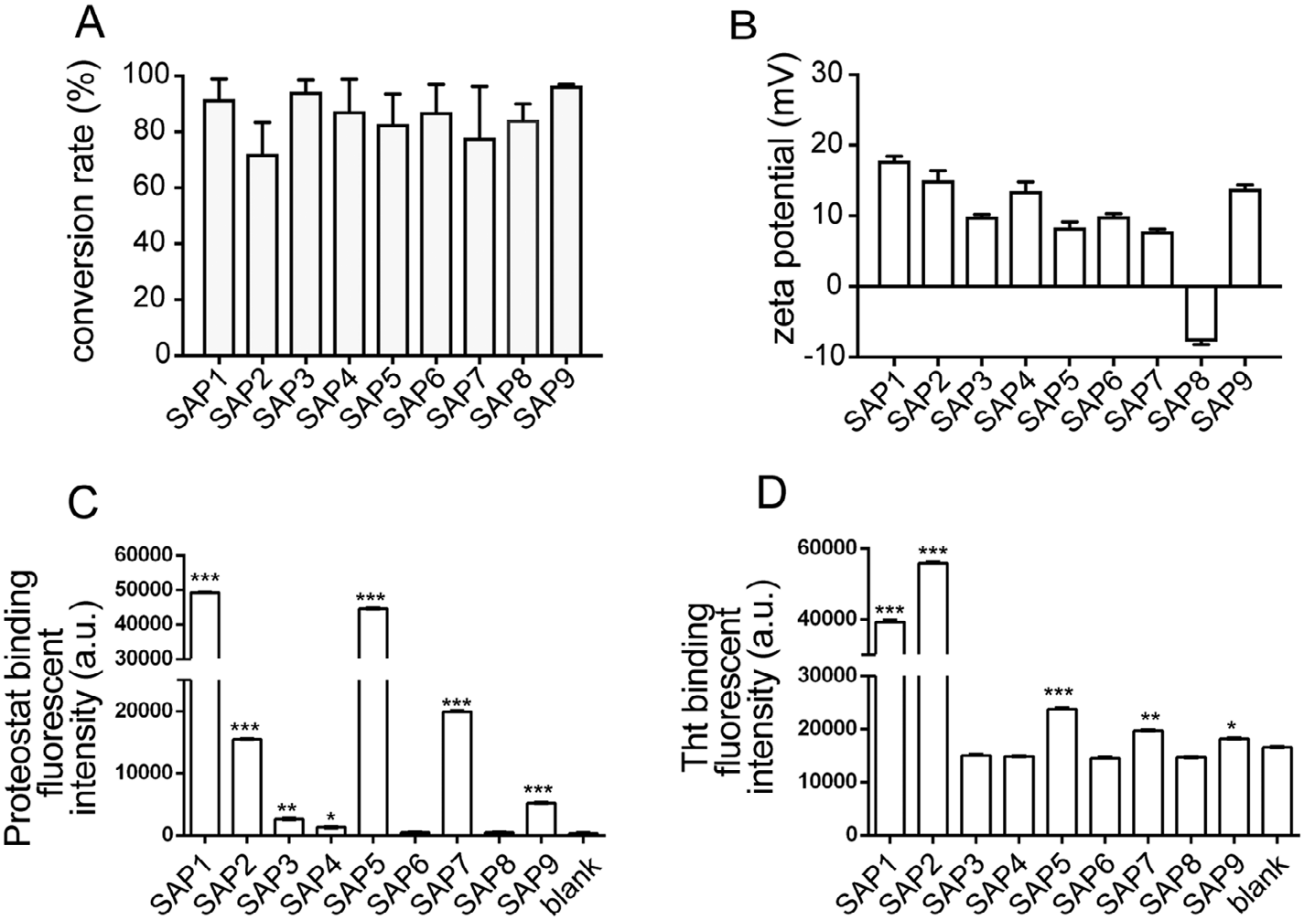
Bio‐physical characterization of SAP‐derived PNFs. (A) Quantification of the conversion rates of SAP1‐SAP9 into PNFs. (B) Analysis of the zeta potential of all SAPs. The zeta potential of all SAPs was positive except for SAP8. (C) Proteostat binding was observed for several SAPs, including SAP1, SAP2, SAP5, SAP7, and SAP9. (D) Several SAPs, including SAP1, SAP2, SAP5, SAP7, and SAP9, were bound by ThT. For statistical analysis in (C, D), one‐way ANOVA was performed, and significance was calculated in relation to blank (^*^, ^**^, ^***^ reflecting *P* ≤ 0.05, 0.01, and 0.001, respectively). All significant changes were indicated; otherwise, no significant changes were obtained.

**FIGURE 2:**
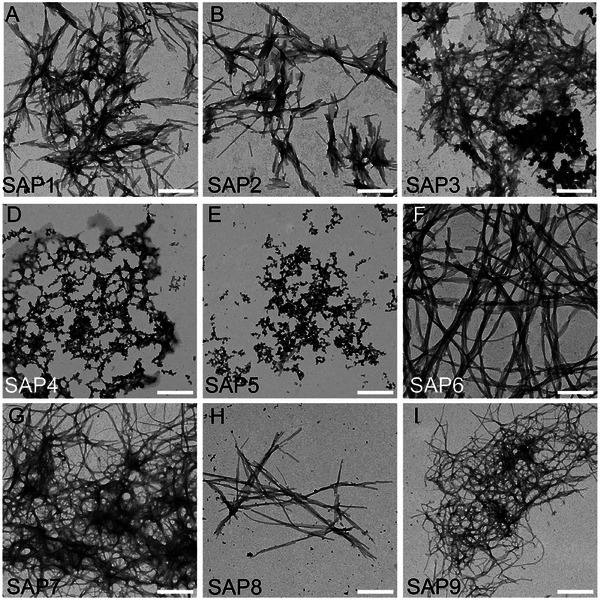
Structural characterization of SAP‐derived PNFs. Representative TEM pictures of all nine SAPs. SAP1 (A), SAP2 (B), SAP3 (C), SAP6 (F), SAP7 (G), SAP8 (H), and SAP9 (I) produced PNF networks consisting of nanofibers of different lengths. In contrast, SAP4 (D) and SAP5 (E) did not assemble into PNFs. Scale‐bar (A–I) = 400 nm.

**FIGURE 3:**
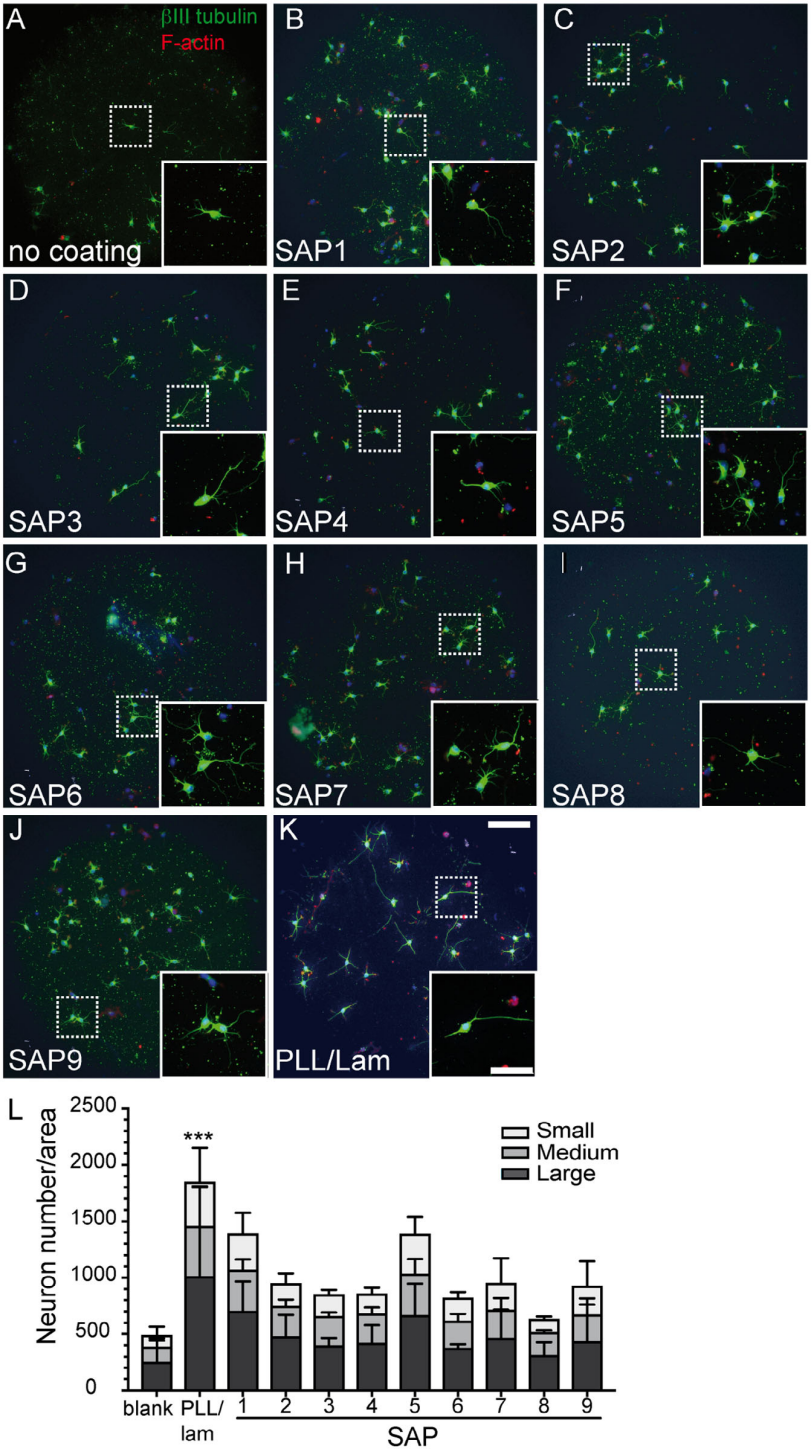
SAPs stimulate the growth of primary mouse neurons. Mouse hippocampal neurons were plated on coverslips without coating (A), coated with SAPs1‐9 (BJ), or PLL/lam (K). After 24 h in culture, neurons were stained for βIII tubulin (green), Phalloidin (red) to label F‐actin, and DAPI (blue). Inserts reveal higher magnifications of dashed boxes. (A) Uncoated coverslips (blank) did not provide a growth substrate for neurons. (B‐J) Coating coverslips with the different SAPs resulted in neuronal growth to a variable extent (quantified in L). (K) PLL and laminin were employed as a favorable growth substrate, resulting in neuronal growth. (L) Quantification of neuron numbers/area according to three different neuron sizes (small, medium, large). N‐numbers included 3 separate experiments consisting of 3–6 pooled animals per experiment. For statistical analysis, Kruskal‐Wallis and Dunn's multiple comparisons test were performed. All significant changes were indicated; otherwise, no significant changes were obtained. Significance was calculated in relation to blank (^*^, ^**^, ^***^ reflecting P ≤ 0.05, 0.01, and 0.001, respectively). Scale‐bar (A–J) = 100 µm; (inserts) = 50 µm.

In this study, we aimed to study novel hybrid nanofibers composed of a SAP backbone conjugated with GF‐derived peptides. Initially, we characterized the physical properties of nine different SAP backbones (Figures 1 and 2), followed by assessment of their biological potential to stimulate neuronal growth (Figure 3). The nine backbones included four previously tested SAPs (SAP1, SAP2, SAP3, and SAP9; [21]) and five novel SAPs (Table 1).

The CR of peptide monomers into assemblies was between 71% and 96% (Figure 1A, Table 2). This indicates robust fiber formation with only a small fraction of peptides remaining as monomers. Previously, we reported positive zeta potentials of SAPs correlating with a higher potential to stimulate neuronal growth [21]. Amongst the nine SAPs tested, eight showed a positive zeta potential (7.9‐17.9 mV) and only one, SAP8, had a negative value (Figure 1B, Table 2). We determined the secondary structure by IR and observed that all SAPs, except for SAP4, contained alpha helices and beta sheets (Figure S1; Table 2) [22]. Finally, the amyloid‐specific dyes thioflavin Proteostat (Figure 1C) and ThT (Figure 1D) were employed to measure SAP aggregation. Five peptides including SAP 1, SAP2, SAP5, SAP7 and SAP9 bound both dyes whereas the other peptides (SAP3, SAP4, SAP6 and SAP8) were negative (summarized in Table 2).

**TABLE 2:** Analysis of biophysical properties of SAPs employed.

Abbreviation	Fibril structure	Zeta potential (mV)	IR spectra	Conversion rate (%)	Proteostat activity	ThT assay
SAP1	+ + + − − + + + +	17.9 ± 0.5	α+β	91	+	+
SAP2		15.1 ± 1.3	α+β	72	+	+
SAP3		10.0 ± 0.2	α+β	94	−	−
SAP4		13.6 ± 1.2	α	87	−	−
SAP5		8.5 ± 0.3	α+β	83	+	+
SAP6		10.0 ± 0.3	α+β	87	−	−
SAP7		7.9 ± 0.3	α+β	78	+	+
SAP8		−7.8 ± 0.4	α+β	84	−	−
SAP9		13.9 ± 0.5	α+β	96	+	+

+: Positive or Active, −: Negative or Non‐active.

Next, fiber assembly and morphology were analyzed by TEM (Figure 2). The majority of the nine SAPs resulted in robust PNF formation with long fibrils engaged in fiber networks (Figure 2). Two of the nine SAPs, SAP4 and SAP5, only formed short PNFs, which accumulated in clusters (Figure 2D,E and Table 2).

Above, we identified several SAPs resulting in PNF networks resembling the extracellular matrix, which might serve as an adhesive and growth substrate for neurons. Thus, primary mouse hippocampal neurons were cultured on glass coverslips coated with PNF networks derived from all nine SAPs (Figure 3). As negative and positive growth substrates, no coating (blank) and PLL/lam (poly‐L‐lysine/laminin), an established growth‐promoting substrate, were used, respectively (Figure 3A,K). Neurons were stained with antibodies directed against βIII tubulin (green) and with phalloidin to label tubulin localization in axons and F‐actin in growth cones, respectively. Neuronal growth was quantified by categorizing neurons according to size (small, medium, and large), and the numbers of neurons/area falling into these three categories were provided (Figure 3L).

**FIGURE 4:**
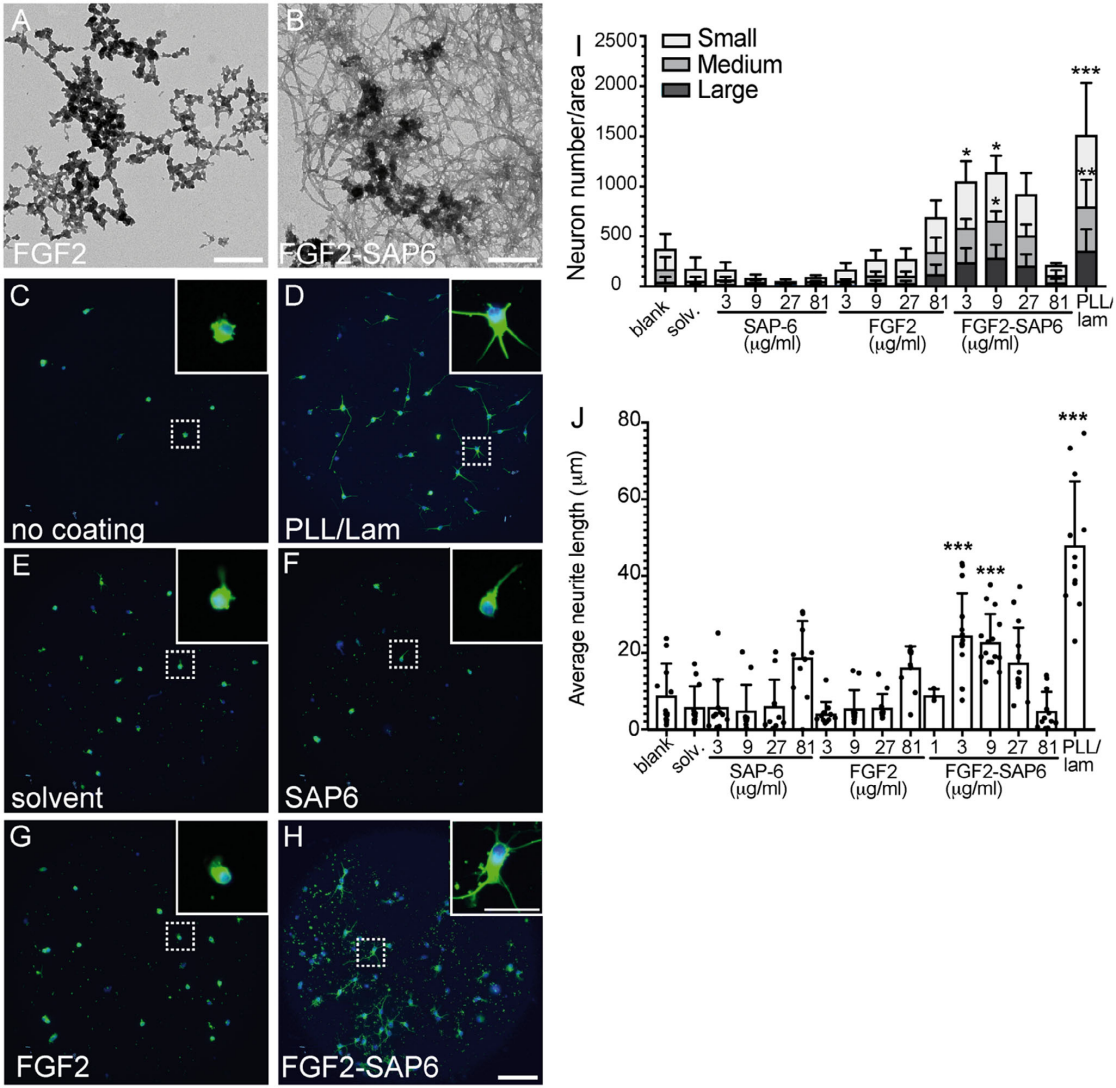
FGF2‐SAP6 derived PNFs stimulate neuronal growth. (A, B) TEM pictures of FGF2 (A) and FGF2‐SAP6 (B), where FGF2 did not form PNFs, but FGF2‐SAP6 did produce PNF networks. (C–H) Mouse hippocampal neurons were plated on uncoated coverslips (C), PLL/lam (D), uncoated coverslips with solvent in medium (E), SAP6 (F), or FGF2 (G) alone or the FGF2‐SAP6 hybrid (H) added to the medium. After 24 h in culture, neurons were stained for βIII tubulin (green) and DAPI (blue). Strongest neuronal growth was observed in PLL/lam (D) or FGF2‐SAP6 (H), whereas SAP6 (F) or FGF2 (G) alone were similar to negative controls (C, E). Inserts reveal higher magnifications of dashed boxes. (I, J) Quantification of neuron numbers/area (I) according to three different neuron sizes (small, medium, large) or average neurite length (J). For statistical analysis, a two‐way ANOVA with Tukey's multiple comparisons test was performed, and significance was calculated in relation to blank (^*^, ^**^, ^***^ reflecting *P* ≤ 0.05, 0.01, and 0.001, respectively). N‐numbers included >5 separate experiments. Each experiment consisted of 3–6 pooled animals per experiment. Each dot in (J) represents one coverslip with >50‐100 neurons/coverslip analyzed. For statistical analysis, an ordinary one‐way ANOVA and Tukey's multiple comparisons test were performed, and significance was calculated in relation to solvent (^*^, ^**^, ^***^ reflecting *P* ≤ 0.05, 0.01, and 0.001, respectively). All significant changes were indicated otherwise no significant changes were obtained. Scale‐bar (A, B) = 400 nm; (C–H) = 100 µm; (inserts) = 30 µm.

**FIGURE 5:**
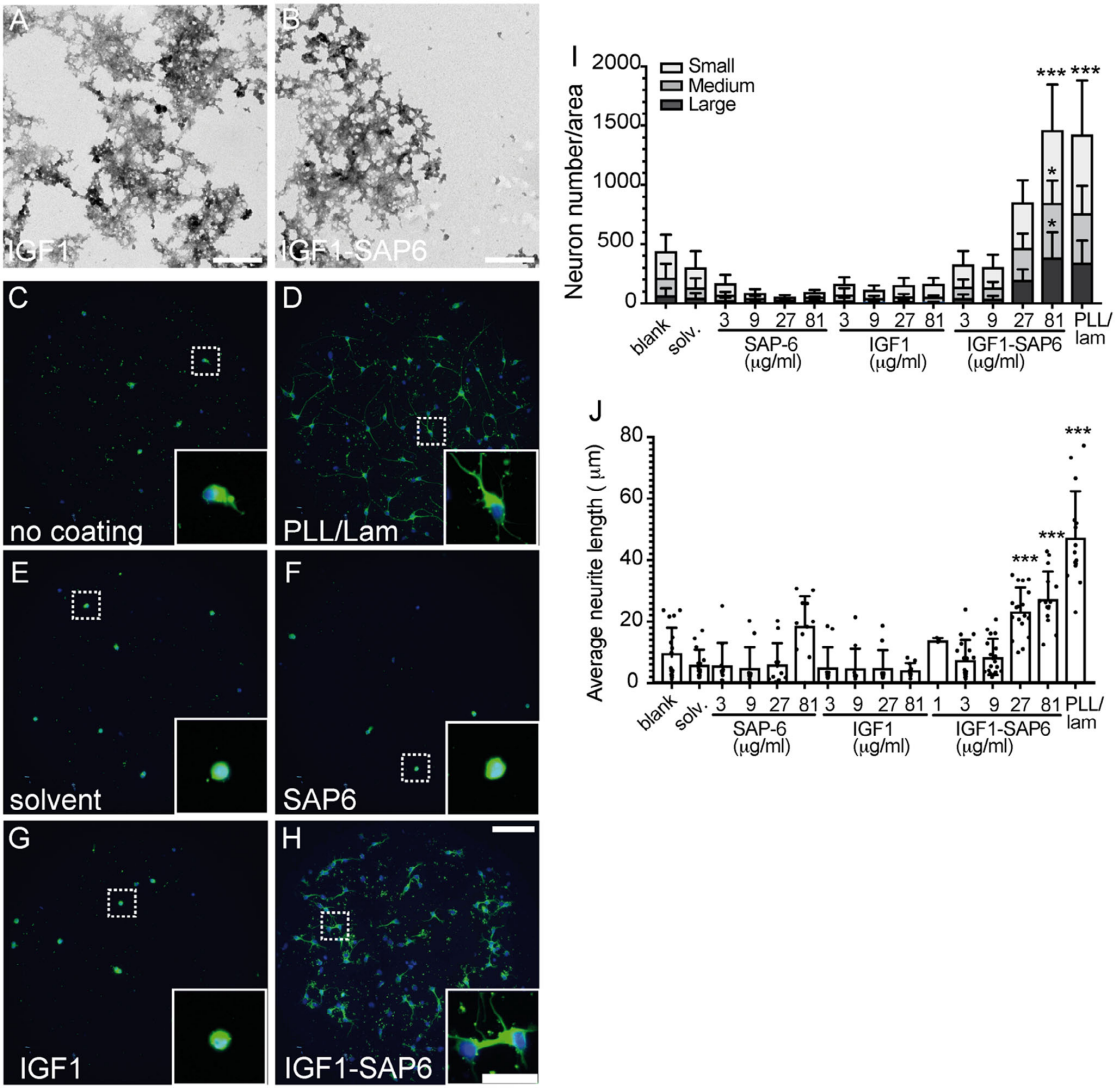
PNFs derived from IGF1‐SAP6 enhance neuronal growth. (A, B) TEM pictures of IGF1 (A) and IGF1‐SAP6 (B). Both peptides did not produce PNFs networks. (C–H) Mouse hippocampal neurons were plated on uncoated coverslips (C), PLL/lam (D), uncoated coverslips with solvent in medium (E), SAP6 (F), or IGF1 (G) alone or the IGF1‐SAP6 hybrid (H) added to the medium. One day later, neurons were stained for βIII tubulin (green) and DAPI (blue). Strongest neuronal growth was obtained in PLL/lam (D) or IGF1‐SAP6 (H). In contrast, SAP6 (F) or IGF1 (G) alone were similar to negative controls (C, E). Inserts reveal higher magnifications of dashed boxes. (I, J) Quantification of neuron numbers/area (I) according to three different neuron sizes (small, medium, large) or neurite length (J). N‐numbers included >5 individual experiments. Each experiment consisted of 3–6 pooled animals per experiment. Each dot in (J) represents one coverslip with >50‐100 neurons/coverslip analyzed. For statistical analysis, an ordinary one‐way ANOVA and Tukey's multiple comparisons test was performed and significance was calculated in relation to solvent (^*^, ^**^, ^***^ reflecting *P* ≤ 0.05, 0.01 and 0.001, respectively). All significant changes were indicated otherwise no significant changes were obtained. Scale‐bar (A, B) = 400 nm; (C–H) = 100 µm; (inserts) = 30 µm.

As expected, neurons cultured on uncoated coverslips resulted in sparse neuronal growth with regard to all three categories of neuron size (Figure 3A,L). All SAPs employed as growth substrates resulted in better neuronal outgrowth (Figure 3B–K) compared to the uncoated condition (Figure 3K). This included SAP1, which was previously reported for its strong neuronal growth activity [21] and was also one of the strongest growth stimulators in this study (Figure 3B,L). In contrast, SAP8, which was the only SAP with a negative zeta potential (Figure 1 and Table 1), resulted in the weakest growth‐promoting activity (Figure 3I,L). Besides SAP8, all other SAPs resulted in at least a two‐fold increase in neuron size and are therefore suitable as backbones for growth factor conjugation (see below). Since SAP6 was amongst the SAPs with the shortest amino acid sequence, high conversion rate, positive zeta potential, and long fiber formation (Figures 1, 2, 3; Table 2), we selected SAP6 as the backbone for conjugation with FGF2 and IGF1‐derived peptides (Figures 4 and 5).

### 3.2 FGF2‐SAP Hybrid PNFs Stimulate Neuronal Growth

Members of the FGF family, particularly FGF2, are well‐established activators of neuronal growth [18]. Herein, we tested whether an FGF2 mimic (YRSRKYSSWYVALKR; [7] stimulates primary mouse neuronal growth. The chemical identity and purity of the synthesized FGF mimic and the conjugation of FGF2 to SAP6, abbreviated as FGF2‐SAP6, are shown in (Figure S2a–d). FGF2‐SAP6 resulted in a conversion rate of 99%, with a zeta potential of both the FGF2 mimic alone and FGF2‐SAP6 close to zero (Table 3).

**TABLE 3:** Summary of biophysical properties of hybrid peptides.

Abbreviation	Fibril structure	Zeta potential (mV)	IR spectra	Conversion rate (%)
FGF	−	−0.80 ± 0.7	x	x
FGF‐SAP	−	0.8 ± 0.1	α+β	99
FGF‐SAP_0.6_/SAP_0.3_	+			
FGF‐SAP_0.3_/SAP_0.6_	+			
FGF‐SAP_0.1_/SAP_0.9_	+			
IGF	−	−1.8 ± 1.4	x	x
IGF‐SAP	−	−0.6 ± 0.3	α+β	96
IGF‐SAP_0.6_/SAP_0.3_	−			
IGF‐SAP_0.3_/SAP_0.6_	−			
IGF‐SAP_0.1_/SAP_0.9_	+			
FGFSAP_0.1_/IGFSAP_0.4_/SAP_0.5_	+			
FGFSAP_0.25_/IGFSAP_0.25_/SAP_0.5_	+			

+: Positive or Active, −: Negative or Non‐active.

In neuronal growth assays, the FGF2 mimic alone, SAP6 alone, and the FGF2‐SAP6 hybrid peptides were analyzed in two culture conditions: (i) added to the growth medium and (ii) coated as growth substrates on coverslips (Figure S3). In the latter condition, SAP6 alone stimulated neuronal growth as previously observed (Figure 3), whereas the FGF2 mimic alone expectedly adhered poorly to coverslips and resulted in neuronal growth comparable to the uncoated coverslips (Figure S3). FGF2‐SAP6 enhanced neuronal growth but did not exceed the growth activity provided by SAP6 alone (Figure S3).

Therefore, we focused on the application of peptides directly to the growth medium where neurons had to grow on uncoated coverslips (except for the positive control PLL/lam). We used TEM to characterize peptide structures. While the FGF2 mimic alone did not elaborate PNF structures (Figure 4A), the FGF2‐SAP6 hybrid assembled into PNF networks (Figure 4B).

Hippocampal neurons exhibited minimal outgrowth on uncoated coverslips (Figure 4C) and on coverslips incubated with solvent but without peptides (Figure 4E,I,L). In contrast, neurons demonstrated significant growth on a PLL/lam substrate even in the absence of peptide addition (Figure 4D). When added to the growth medium, SAP6 alone added to the growth medium resulted in poor neuronal growth (Figure 4F), in contrast to when it was applied as a coated growth substrate (Figure 3). Only at the highest concentrations, was SAP6 in medium able to augment average neurite length (Figure 4J). Similarly, the FGF2 mimic alone (Figure 4G) increased both the number of neurons exhibiting outgrowth (Figure 4I) and neurite length (Figure 4J), only with the highest concentrations added to the medium. Notably, the FGF2‐SAP6 fusion peptide strongly stimulated neuron growth (Figure 4H). This effect was observed even at the lowest FGF2‐SAP6 concentrations, where individual application of FGF2 mimic or SAP6 alone did not enhance neuron size (Figure 4I) or average neurite length (Figure 4J). In summary, FGF2‐SAP6 formed a PNF structure that allowed for better cell‐surface interactions of cells and activated neuronal growth in vitro.

### 3.3 IGF1‐SAP6 Enhances Primary Mouse Neuron Growth

Full‐length IGF1 is a potent growth stimulator of a wide range of neurons [17]. Here, we analyzed whether the 12‐amino acid IGF1 peptide sequence (see Table 2), derived from full‐length IGF1, can stimulate neuronal growth of primary neurons (Figure 5) as previously observed in neural stem cells [10]. As above (Figure 4), the IGF1 mimic was conjugated with SAP6, and the activity of the resulting IGF1‐SAP6 hybrid was compared to its single constituents (Figure 5). The chemical identity and purity of the synthesized IGF mimic and IGF1‐SAP6 are presented in (Figure S2e–h). IGF1‐SAP6 exhibited a high conversion rate of 96% (Table 3). The zeta potential of the IGF1 peptide alone was −1.8 ± 1.4 mV, which was shifted toward a slight positive value of −0.6 ± 0.3 mV upon conjugation with SAP6 for IGF1‐SAP6 (Table 3).

First, IGF1‐SAP6 was used to coat coverslips as described above, and enhanced neurite growth compared to both blank and IGF1 mimic alone. However, as observed with FGF2‐SAP6 (Figure S4), IGF1‐SAP6 was not able to further enhance neurite growth compared to SAP6 alone under these conditions (Figure S4).

Subsequently, IGF1‐SAP6 was also analyzed when added to the growth medium (Figure 5). IGF1 mimic alone did not form PNFs (Figure 5A). Similarly, conjugation of IGF1 to SAP6 did not result in PNF network formation (Figure 5B). As noted with FGF2‐SAP experiments above, IGF1‐SAP6 (Figure 5H) exhibited a concentration‐dependent positive effect on neuron size (Figure 5I) and average neurite length (Figure 5J). Quantification revealed that the addition of IGF1‐SAP6 to the growth medium without any coating of the coverslips (Figure 5H) achieved neuronal growth comparable to the positive control, where coverslips were coated with PLL/lam (Figure 5D; quantified in Figure 5I,J). In contrast, the individual components of IGF1‐SAP6, i.e., SAP6 (Figure 5F) and IGF1 (Figure 5G), when added individually to the growth medium, did not improve neuronal growth beyond negative controls (uncoated coverslips ± solvent; Figure 5C,E).

Thus, an IGF mimic peptide fused with SAP sequences produced robust primary neuron growth and outperformed its individual constituting sequences.

### 3.4 Analysis of PNFs Composed of FGF2‐SAP and IGF1‐SAP

**FIGURE 6:**
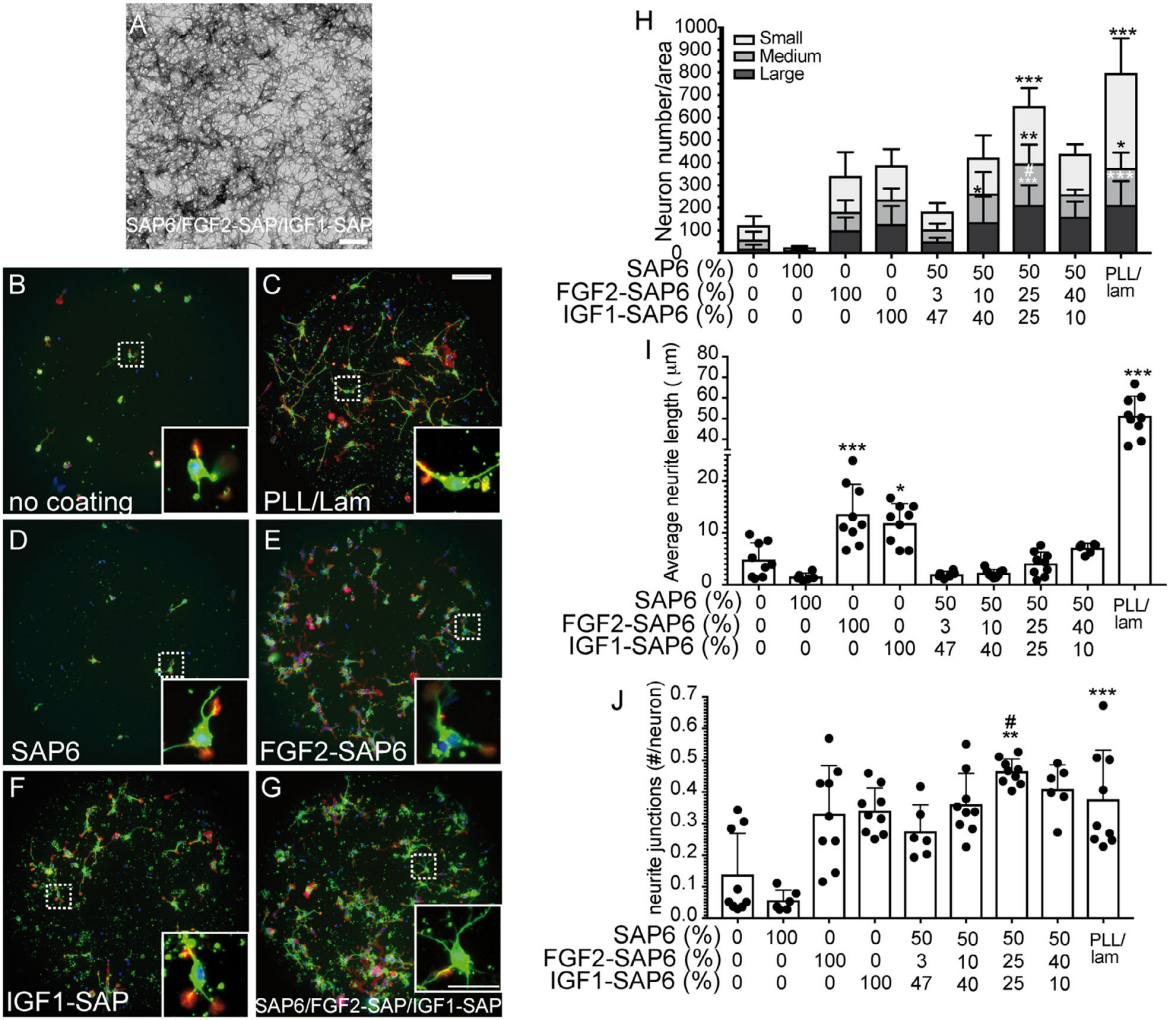
Specific mixtures of FGF2‐SAP6 and IGF1‐SAP6 with SAP6 form growth‐enhancing PNFs. (A) TEM picture of PNFs composed of SAP6 (50%), FGF2‐SAP6 (25%), and IGF1‐SAP6 (25%). (B–G) Mouse hippocampal neurons were plated on uncoated coverslips (B), PLL/lam (C), or uncoated coverslips with SAP6 (D), FGF2‐SAP6 (E), IGF1‐SAP6 (F), or SAP6/FGF2‐SAP6/IGF1‐SAP6 (G) added to the medium. Neurons were labeled for βIII tubulin (green), F‐actin (red), and DAPI (blue). Four conditions, including PLL/lam (C), FGF2‐SAP6 (E), IGF1‐SAP6 (F), and SAP6/FGF2‐SAP6/IGF1‐SAP6 (G), showed the strongest neuronal growth, superior to no coating (B) and SAP6 alone (D). Inserts reveal higher magnifications of dashed boxes. (H–J) Quantification of neuron numbers/area (H) according to three different neuron sizes (small, medium, large), neurite length (I), and the number of junctions where two neurites branch off (J). N‐numbers included individual experiments. Each experiment consisted of 3–6 pooled animals per experiment. Each dot in (I, J) represents one coverslip with >50–100 neurons/coverslip analyzed. For statistical analysis, a Kruskal‐Wallis with Dunn's multiple comparisons test was performed, and significance was calculated in relation to blank (^*^, ^**^, ^***^ reflecting P ≤ 0.05, 0.01, and 0.001, respectively, or # in relation to FGF2‐SAP6). All significant changes were indicated; otherwise, no significant changes were obtained. Scale‐bar (A) = 400 nm; (B–G) = 100 µm; (inserts) = 30 µm.

It is widely accepted that GF cocktails often have superior activity in promoting neuronal growth and regeneration compared to a single application (e.g. [12]). PNFs composed of several GFs may serve as suitable bioactive molecules by providing such combined GF activity. So far, several studies have analyzed PNF‐derived hydrogels that release GFs in defined temporal windows to modulate cellular processes [13, 14, 15]. Herein, we fused GFs to SAP6 and generated PNFs stably presenting FGF2 and IGF1 mimetic peptides to neurons. Due to the fusion with the SAP backbone, GFs were not released to the environment, potentially enabling a more sustained cellular activity (Figure 6).

In order to analyze PNFs with FGF and IGF peptides, SAP6 abundance was kept at 50% of the final solution, and this was increased to 100% by different combinations of FGF2‐SAP6 and IGF1‐SAP6 (Figure 6). After incubation, the mixtures produced PNFs (Figure 6A) similar to PNFs composed of FGF2‐SAP (Figure 4) or IGF1‐SAP6 (Figure 5) alone. All molecules were added to the growth medium of primary mouse hippocampal neurons as before (Figures 4 and 5). FGF2‐SAP6 (Figure 6D) and IGF1‐SAP6 (Figure 6E) significantly stimulated the number of neurons with growth (Figure 6H), as well as average neurite length (Figure 6I) and number of neurite junctions (Figure 6J) in accordance with previous findings (Figures 4 and 5).

For the combined PNFs composed of both FGF2‐SAP6 and IGF1‐SAP6 (Figure 6F), we observed that a mixture containing 50% SAP6, 25% FGF2‐SAP6 and 25% IGF1‐SAP6 resulted in the most promising effects. For this combination, neuron numbers/area (Figure 6H) and branching (Figure 6J) but not neurite length (Figure 6I) were stimulated compared to a single application. In contrast, other combinations were less efficient and resulted in lower growth parameters compared to individual application (Figure 6H–J).

In summary, PNFs with combined growth factor activity could be identified for neuronal growth.

## 4 Discussion

Tissue development, function, and regeneration are orchestrated by specific GF combinations. In this study, we described a nanofiber‐based approach to incorporate desired GF‐mimetic sequence combinations into PNF networks, which might be well‐suited for tissue regeneration. For this, peptides derived from two well‐established, pro‐regenerative GFs, FGF2 and IGF1, were connected to an SAP backbone. Such fusion peptides forming PNF networks were able to modulate cellular growth, as demonstrated by primary mouse hippocampal neurons in this study. Our findings are in line with previous reports employing different GFs, including BDNF (brain‐derived neurotrophic factor) or NGF (nerve growth factor), which likewise stimulated neuronal growth or regeneration parameters [23, 24].

The structure of SAPs is governed by the intrinsic amino acid sequence, including the natural propensities of individual residues, charge distribution, and aromatic interactions [25, 26, 27]. External factors such as salts or buffers, pH, and temperature are also known to influence SAP structure [27, 28]. In addition, sample processing conditions—specifically the order in which these external factors are applied to the peptides—can play an important role in determining the resulting SAP structures [29]. In the case of SAPs for neuronal repair, the first prerequisite for growth stimulation appeared to be a positive charge, beta‐sheet formation, and the emergence of a PNF network introduced by the SAP amino acid sequence [21]. Interestingly, SAP4, where the N‐terminus of SAP3 had been extended with WWWQ, results in the disappearance of the beta‐sheet, only presenting an α‐helical signal at 1672 cm^−1^ in the IR spectrum. This might be explained by the interaction between hydrogen bonds and the geometry of the peptide backbone and TFA [30]. Furthermore, the PNFs seen for SAP3 are replaced by ill‐defined aggregates for SAP4 (Figure 2). Additionally, SAP8 was the only sequence containing His (H) and the only assembly with a negative zeta potential, likely responsible for its poor support of neuronal cells (Figure 3). Unlike the aliphatic amine group in Lys (K, pKa = 10.5), the pKa value of the imidazole group in His is about 6.0. At physiological pH, the side chains of histidine are only partially protonated, resulting in each histidine residue carrying less than one unit of positive charge in peptides. Thus, direct replacement of Lys with His significantly decreased the overall positive charges [31]. Additionally, the zeta potential of a His‐containing peptide sequence is not only pH‐dependent but also dependent on the molecular interaction and folding behavior in the sequence [32, 33].

In line with a previous report [21], only SAPs with a positive but not negative (SAP8; Figures 1, 2, 3) zeta potential were able to promote cell adhesion and growth. Thus, close interaction between positively charged PNFs and the negatively charged plasma membrane appears to underlie the growth‐promoting SAP activity. Second, SAP backbones, when covalently connected to GF peptides, augment the biological activity of GF derived peptides compared to their individual constituents (Figures 4 and 5). Herein, this was shown by both FGF2‐SAP6 and IGF1‐SAP6, which had a higher neuronal growth potential compared to SAP alone and single GF application. Notably, FGF2‐SAP6 (Figure 4) but not IGF1‐SAP6 (Figure 5) formed PNF networks. This correlated with their potential to increase neuronal growth, which was enhanced by both conjugates. However, IGF1‐SAP6 required higher concentrations compared to FGF2‐SAP6 to stimulate neuronal growth (Figures 4 and 5).

Such PNF scaffolds resemble extracellular matrix (ECM) structures. Indeed, it is known that GF interaction with ECM components, such as proteoglycans, raises local GF concentration, modulates receptor activation, and influences downstream signal propagation in cells [34]. Therefore, GF‐SAP fusion peptides forming ECM‐like structures might have stronger signaling potential compared to soluble GF concentrations. Furthermore, SAP‐derived nanofibers provide a growth substrate with defined stiffness. Previously, mechanical ECM properties such as substrate stiffness were shown to affect neuronal growth, morphology, and development [35]. Indeed, both we and others have reported that increased substrate stiffness enhances neuronal growth [21, 36]. Thus, it is conceivable that GF‐conjugated SAPs such as FGF2‐SAP6 and IGF1‐SAP6 have an impact on neuronal growth parameters through modulating substrate mechanics.

In this study, we present an approach that enables the combination of different bioactive molecules, such as GF‐derived peptides, into nanofibers. Previously, GF combinations were released by hydrogels implanted in injured tissues, which were shown to have beneficial effects for tissue regeneration, including regeneration of the injured spinal cord in animal models [10]. However, hydrogel stability and the temporal parameters of GF release are difficult to control in living organisms. Herein, bioactive molecules were covalently bound to the SAP, thereby forming PNFs, which prevent the release of GF molecules from the fibers. Such PNFs, when introduced to injury sites in vivo, may result in higher local GF concentrations sustained over longer periods of time, which might improve their activity. In a previous report, we have shown that SAP‐derived PNFs adhere to injury sites for up to three weeks [21], covering a substantial time span for stimulating repair processes. Thus, although in vivo validation is missing, such stable GF‐presenting PNFs might represent a valuable therapeutic approach to be further tested in animal models. Of note, for therapeutic purposes, stability of SAP‐derived peptides can be further enhanced through chemical modifications, including lipidation [37]. A further advantage with regard to clinical application and production costs is the low molecular weight of the fusion peptides used in the current experiments (approx. 20 aa) when compared to the full‐length GF sequence (approx. 70–150 aa).

Finally, since SAP backbones such as SAP6 presented in this study can be functionalized with two (or more) GF‐derived peptides or other bioactive peptides, it is possible to devise tissue‐specific combinations of molecules for application to injured tissues. In this study, the first combinatory approaches in such a direction were presented. However, more detailed work on SAP and GF mixtures is necessary to optimize their activity and demonstrate functional relevance in animal injury models.

## Author Contributions

B.K. and C.V.S. conceived the study and supervised the project. K.R., Y.L.T., and N.J. performed experiments and data analysis. B.K. prepared figures and drafted the manuscript. All authors read and approved the manuscript.

## Conflicts of Interest

The authors declare no conflicts of interest.

## Ethics Approval and Consent to Participate

All experimental procedures were approved by the regional authority (Regierungspräsidium Tübingen, Germany).

## Supporting information




**Supporting File**: mabi70148‐sup‐0001‐SuppMat.pdf.

## Data Availability

The data that support the findings of this study are available from the corresponding author upon reasonable request.
