# Supplementary material for: Identification of Novel Growth Factor Conjugated Nanofibers for Stimulation of Neuronal Growth
Source: Macromol Biosci. 2026 Jan 23;26(1):e00585. doi: 10.1002/mabi.202500585 (PMC12829522; doi:10.1002/mabi.202500585)
Supplement: Supplementary file 1 — Supporting File: mabi70148‐sup‐0001‐SuppMat.pdf. [file MABI-26-e00585-s001.pdf]

Supporting information

Identification of novel growth factor conjugated nanofibers for stimulation of neuronal growth

Yu-Liang Tsai[1#], Karla Rivera[2#], Nayeong Jeon[1], Bernd Knöll[2*] and Christopher V. Synatschke[1*]

[1] Max Planck Institute for Polymer Research
Ackermannweg 10
55128 Mainz
Germany

[2]Institute of Neurobiochemistry
Ulm University
Albert-Einstein-Allee 11
89081 Ulm
Germany

# shared first authors

* Corresponding authors
Christopher V. Synatschke and Bernd Knöll
synatschke@mpip-mainz.mpg.de
bernd.knoell@uni-ulm.de

The authors declare no competing financial interests.

Table of contents

Figure S1. IR spectra of 9 SAP backbones

Figure S2. Chemical identity and purity of synthesized peptides.

Figure S3. Coated FGF2-SAP6 enhances neuronal outgrowth.

Figure S4. IGF1-SAP6 coating promotes neuron growth.

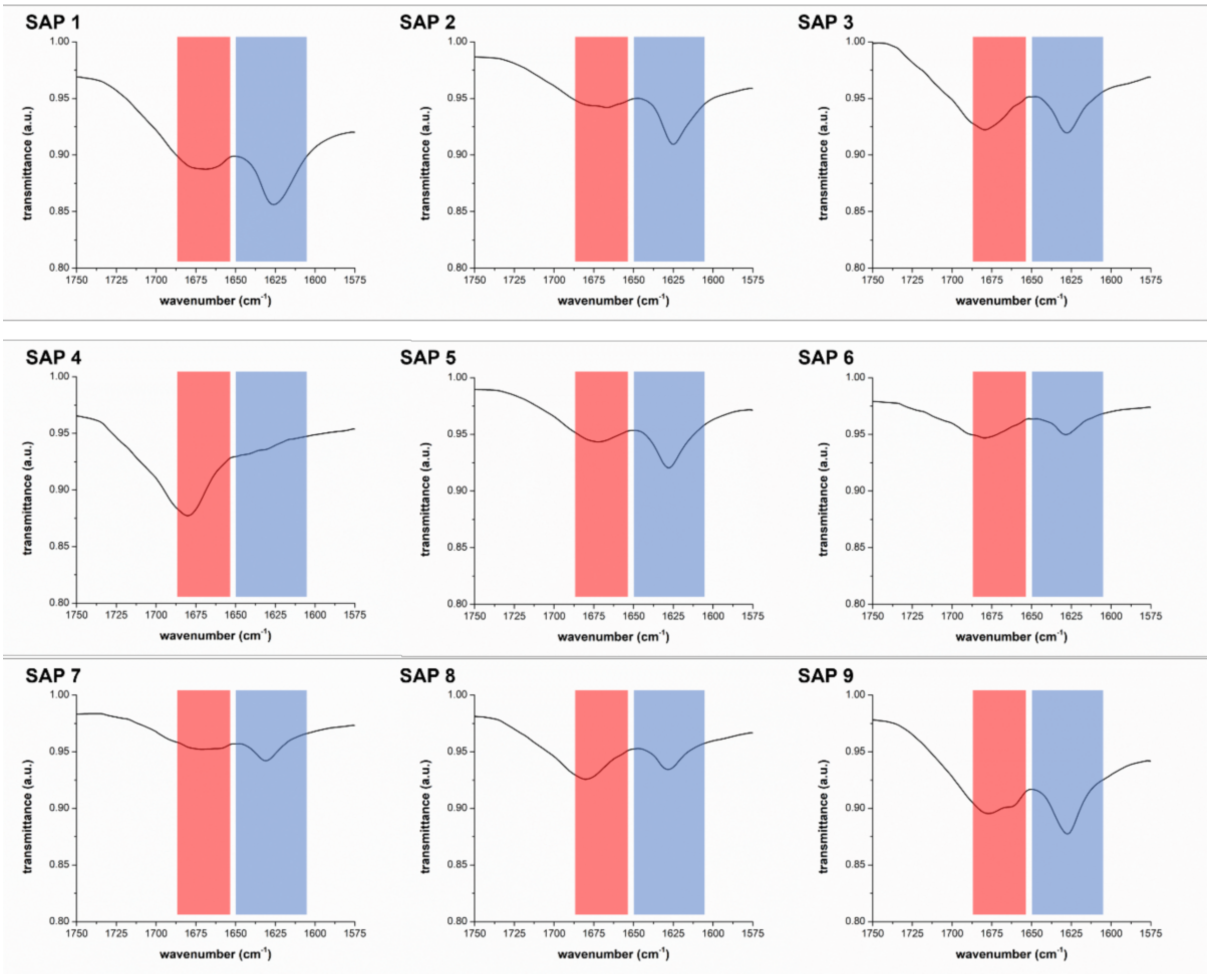

Figure S1

IR spectra of the nine SAP backbones.

IR spectra of SAP1 to SAP9. The blue area represents β-sheet structure and the red area stands for α-helix structure.

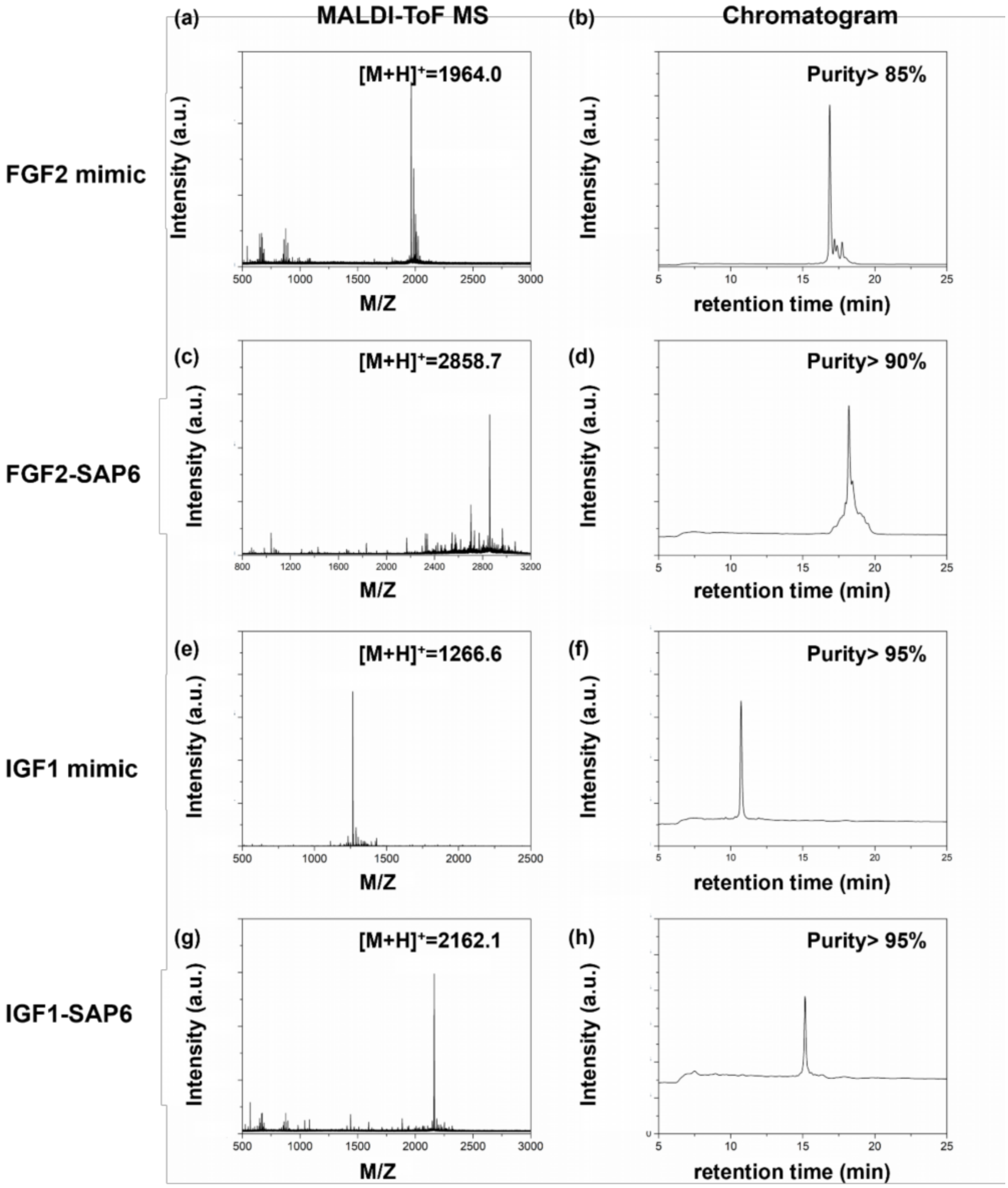

Figure S2

Chemical identity and purity of synthesized peptides

(a) [M+H]$^+$ = 1964.0 was detected for FGF2 mimic (theoretical MW = 1963.3). (b) HPLC trace of FGF2 mimic with a retention time (t_R) = 16.6–16.9 min. (c) [M+H]$^+$ = 2858.7 was detected for FGF2-SAP6 (theoretical MW =2858.4). (d) HPLC trace of FGF2-SAP6 with a retention time (t_R) = 17.3–17.6 min. (e) [M+H]$^+$ = 1266.6 was detected for IGF1 mimic (theoretical MW = 1266.3). (f) HPLC trace of IGF1 mimic with a retention time (t_R) = 10.3–10.5 min. (g) [M+H]$^+$ = 2162.1 was detected for IGF1-SAP6 (theoretical MW = 2161.5). (h) HPLC trace of IGF1-SAP6 with a retention time (t_R) = 15.0–15.3 min.

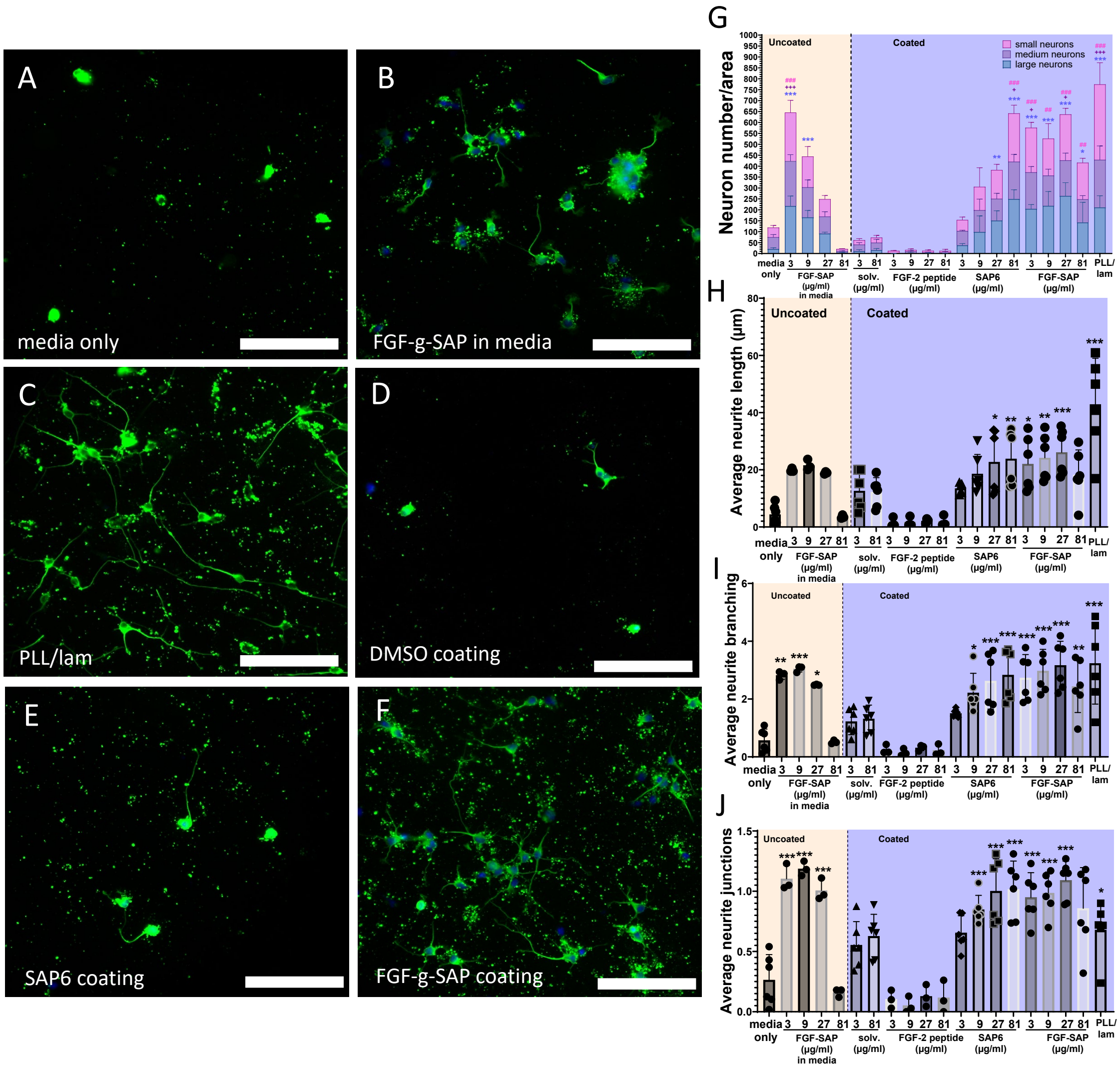

Figure S3

Coated FGF2-SAP6 enhances neuronal outgrowth

(A-F) Representative images of mouse hippocampal neurons plated on either uncoated coverslips with (A) normal medium or (B) medium containing FGF2-SAP6, or on coverslips coated with (C) PLL/lam, (D) DMSO, (E) SAP6 or (F) FGF2-SAP6. All images correspond to 3 µg/mL peptide coating. Neurons were grown for 24 hours and stained for βIII tubulin (green) and DAPI (blue). Neurons grown on uncoated coverslips with medium only (A) and neurons grown on coverslips coated with DMSO (D) show poor outgrowth. Coating with 3 µg/mL of SAP6 resulted in a mild, statistically insignificant increase in neuronal numbers and neurite length. In contrast, when FGF2-SAP was either added to the medium (B) or used to coat coverslips (F), a significant increase in neuronal numbers, neurite length, branches and junctions was observed. (G) Quantification of neuron areas per FOV, categorized as small, medium and large neurons. (H) Average neurite length per neuron (µm). (I) Average neurite branching per neuron per condition. (J) Average number of neurite junctions per neuron. N-numbers represent 3 independent experiments, consisting of 3-6 pooled animals per experiment. Each data point represents one coverslip with >100-500 neurons analyzed per coverslip. Statistical analysis was performed using an ordinary one-way ANOVA and Brown-Forsythe test. Significance was calculated in relation to the blank control (*, **, *** reflecting p ≤ 0.05, 0.01 and 0.001, respectively). Scale bar = 100µm.

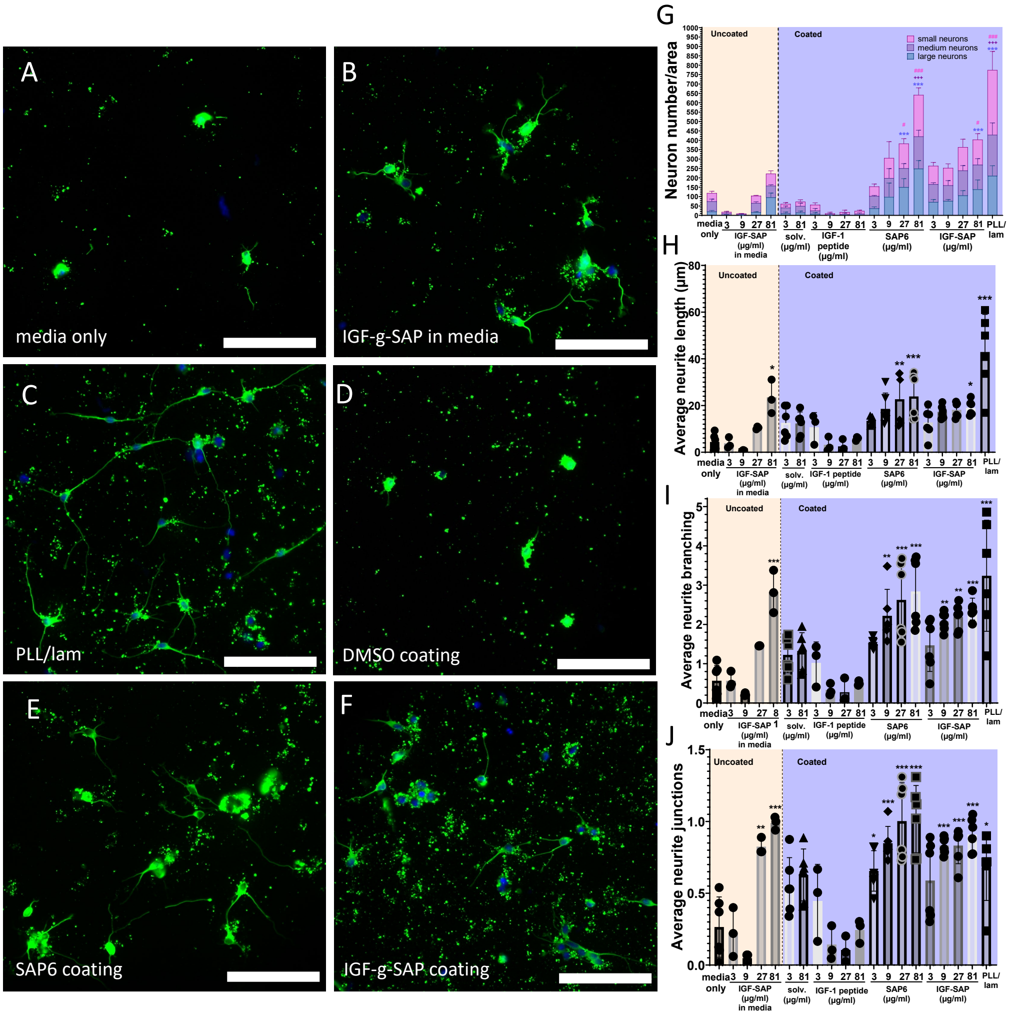

Figure S4

IGF1-SAP6 coating promotes neuron growth

(A–F) Representative fluorescence images of primary mouse hippocampal neurons cultured on (A) uncoated coverslips in standard medium, (B) uncoated coverslips with IGF1-SAP6 added to the medium, or coverslips coated with (C) PLL/Lam, (D) DMSO, (E) SAP6, or (F) IGF1-SAP6 (81 µg/mL for all coatings). Neurons were maintained for 24 hours and immunostained for βIII tubulin (green) and DAPI (blue). Cultures grown on uncoated or DMSO-coated surfaces displayed sparse neurons with limited neurite extension. SAP6-coated coverslips (E), medium-supplemented (B) and surface-bound (F) IGF1-SAP6 conditions markedly enhanced neuronal density, neurite elongation, and overall network complexity. (G) Distribution of neuronal sizes per field of view (FOV), categorized as small, medium, or large. (H) Mean neurite length per neuron (µm). (I) Quantification of neurite branching per neuron. (J) Total number of neurite junctions per neuron across conditions. Data are based on three independent experiments with 3–6 pooled animals each. Each point denotes one coverslip, encompassing >100–500 neurons analyzed. Statistical comparisons were performed using an ordinary one-way ANOVA with Brown–Forsythe correction. Significance was calculated relative to the blank control (*, **, *** corresponding to $p \leq 0.05$, $p \leq 0.01$, and $p \leq 0.001$, respectively). Scale bar = 100 µm
